# The Effectiveness of Unilateral Cochlear Implantation on Performance-Based and Patient-Reported Outcome Measures in Finnish Recipients

**DOI:** 10.3389/fnins.2022.786939

**Published:** 2022-06-06

**Authors:** Aarno Dietz, Antje Heinrich, Timo Törmäkangas, Matti Iso-Mustajärvi, Petrus Miettinen, Tytti Willberg, Pia H. Linder

**Affiliations:** ^1^Department of Otolaryngology, Kuopio University Hospital, Kuopio, Finland; ^2^Division of Human Communication, Development and Hearing, School of Health Sciences, University of Manchester, Manchester, United Kingdom; ^3^Department of Health Sciences, University of Jyväskylä, Jyväskylä, Finland; ^4^Faculty of Health Sciences, University of Eastern Finland, Kuopio, Finland; ^5^Department of Otorhinolaryngology – Head and Neck Surgery, Turku University Hospital, Turku, Finland

**Keywords:** cochlear implant, outcome measures, Quality of Life, SSQ, NCIQ, speech perception

## Abstract

Understanding speech is essential for adequate social interaction, and its functioning affects health, wellbeing, and quality of life (QoL). Untreated hearing loss (HL) is associated with reduced social activity, depression and cognitive decline. Severe and profound HL is routinely rehabilitated with cochlear implantation. The success of treatment is mostly assessed by performance-based outcome measures such as speech perception. The ultimate goal of cochlear implantation, however, is to improve the patient’s QoL. Therefore, patient-reported outcomes measures (PROMs) would be clinically valuable as they assess subjective benefits and overall effectiveness of treatment. The aim of this study was to assess the patient-reported benefits of unilateral cochlear implantation in an unselected Finnish patient cohort of patients with bilateral HL. The study design was a prospective evaluation of 118 patients. The patient cohort was longitudinally followed up with repeated within-subject measurements preoperatively and at 6 and 12 months postoperatively. The main outcome measures were one performance-based speech-in-noise (SiN) test (Finnish Matrix Sentence Test), and two PROMs [Finnish versions of the Speech, Spatial, Qualities of Hearing questionnaire (SSQ) and the Nijmegen Cochlear Implant Questionnaire (NCIQ)]. The results showed significant average improvements in SiN scores, from +0.8 dB signal-to-noise ratio (SNR) preoperatively to −3.7 and −3.8 dB SNR at 6 and12 month follow-up, respectively. Significant improvements were also found for SSQ and NCIQ scores in all subdomains from the preoperative state to 6 and 12 months after first fitting. No clinically significant improvements were observed in any of the outcome measures between 6 and 12 months. Preoperatively, poor SiN scores were associated with low scoring in several subdomains of the SSQ and NCIQ. Poor preoperative SiN scores and low PROMs scoring were significantly associated with larger postoperative improvements. No significant association was found between SiN scores and PROMs postoperatively. This study demonstrates significant benefits of cochlear implantation in the performance-based and patient-reported outcomes in an unselected patient sample. The lack of association between performance and PROMs scores postoperatively suggests that both capture unique aspects of benefit, highlighting the need to clinically implement PROMs in addition to performance-based measures for a more holistic assessment of treatment benefit.

## Introduction and Purpose of the Study

The ability to understand speech is the most important application of human hearing. Verbal communication enables us to conduct sophisticated social interaction and social relationships, which are essential to our health and wellbeing. Difficulties recognizing speech in the presence of background noise or in multitalker situations is the most common manifestation of hearing loss (HL; Kramer et al., 1998) and may represent a starting point for a gradually progressing social disconnection. It is therefore not surprising that untreated HL is associated with loss of social activities and autonomy, as well as depression and even cognitive decline (Lin et al., 2011; Loughrey et al., 2018). Given its increasing prevalence and serious socioemotional consequences, HL ranks among the greatest public health challenges globally in the coming decades (World Health Organization [WHO], 2021).

Most mild and moderate HL is rehabilitated with conventional hearing aids; severe and profound HL is commonly treated with cochlear implants (CI). In both cases, the primary goal of rehabilitation is to provide a level of verbal communication that enables satisfactory social interaction and performance in most everyday sound environments and situations (i.e., at home, on the phone, in a car, in a restaurant, at work, etc.), thereby improving the patient’s quality of life (QoL).

Predicting the success of cochlear implantation for an individual patient is challenging and requires a holistic approach (Boisvert et al., 2020) beyond measuring aided thresholds, as these do not provide meaningful information about the functional hearing relevant for most everyday hearing situations (Vermiglio et al., 2012). Word and/or sentence perception in quiet have been the most commonly used supra-threshold clinical outcome measures. Numerous studies have shown that cochlear implantation reliably restores sound audibility, thereby enabling speech perception in quiet and non-reverberant surroundings (i.e., sound booth; Gifford et al., 2008; Dietz et al., 2015; Boisvert et al., 2020). However, speech perception tests in quiet are not able to measure functional hearing relevant for most everyday hearing situations. Moreover, speech perception tests in quiet are prone to ceiling effects (Gifford et al., 2008; Dietz et al., 2015). Thus, speech perception tests conducted in background noise are regarded as a more adequate way to measure functionally relevant performance outcomes of cochlear implantation, since background noise better approximates complex listening situations (Holden et al., 2013; Dietz et al., 2015). However, speech-in-noise (SiN) tests have also been criticized for not fully capturing the benefits of cochlear implantation. A more comprehensive assessment may be provided by patient-reported outcome measures (PROMs; McRackan et al., 2019). Although PROMs are often used for hearing aid validation, they are less commonly use for the outcome evaluation of cochlear implantation. This is the case even though PROMs seem well-placed to reflect the impact of the change in hearing performance on a patient’s QoL. PROMs are more holistic than performance-based outcome measures in that they assess not only functional aspects of hearing but also hearing-related socioemotional consequences such as social interaction, self-esteem (SE) and emotional wellbeing (Mertens et al., 2020).

One way to categorize outcome measures is to use the International Classification of Functioning, Disability and Health (ICF; World Health Organsation [WHO], 2001). The ICF classifies health according to three domains, namely an individual’s body function and structure, their activity limitations, and their participation restrictions (World Health Organsation [WHO], 2001). PROMs typically assess activity limitations and/or participation restrictions. In audiological practice these limitations and restrictions are typically tied to communication. Two PROMs of particular importance for the current study are the Nijmegen Cochlear Implant Questionnaire (NCIQ; Hinderink et al., 2000) and the Speech Spatial and Qualities of Hearing scale (SSQ; Gatehouse and Noble, 2004). The NCIQ was specifically developed for CI users and is currently the most commonly used QoL-questionnaire for this patient group. It has been translated into many languages (Sanchez-Cuadrado et al., 2015; Ottaviani et al., 2016; Santos et al., 2017). The NCIQ assesses mainly participation restrictions related to social and emotional aspects of wellbeing. The SSQ is another PROM that has been previously used with CI users, although it has not been fully validated for this population. The SSQ assesses activity limitations related to speech perception, spatial hearing (SH), and sound quality (SQ) for different everyday situations.

In summary, changes in the patient’s activity limitations, participation restrictions, wellbeing, and QoL are rarely assessed in the clinical practice of cochlear implantation, even though these dimensions add unique insights into rehabilitation success beyond performance-based scores.

To provide an objective picture of a patient group, it is paramount to minimize any reporting biases. One way of doing this is to sample patients prospectively without any regard for the success of the intervention or difficulties along the way, and to obtain both pre- and postoperative measures from the same patient. Such a design presents a contrast to many studies that use retrospective and cross-sectional designs where patients are only tested postoperatively. The present study aims to avoid potential reporting bias by using a prospective non-selective longitudinal design that compares the change of QoL, activity limitations, participation restrictions, and SiN perception in adult patients undergoing cochlear implantation.

The primary aim of this study was to investigate performance-based and patient-reported outcome measures (PROMs) after cochlear implantation in an unselected, consecutive Finnish patient cohort undergoing unilateral cochlear implantation. We wanted to understand the benefits of cochlear implantation more fully by investigating the following three questions: (1) what are the changes to communicative ability and QoL in response to cochlear implantation? (2) What is the timeline of change? (3) To what extent do behavioral SiN scores and patient-reported disability scores covary with QoL measures? The ultimate goal was to predict a patient’s rehabilitation success more accurately.

## Materials and Methods

### Study Design

This was a prospective patient cohort study. Patients referred to the Kuopio University Hospital for unilateral cochlear implantation were given the option to participate. According to the institution’s clinical routine, patients were evaluated preoperatively at 2–4 weeks before surgery, at which point they filled out the Finnish NCIQ and the Finnish SSQ. The SiN test was administered within 3 months of the preoperative questionnaire administration and was carried out in the best-aided condition, i.e., according to the device configuration that the patient was using in their everyday life at that point. The postoperative follow-up appointments were scheduled 6 and 12 months after the first fitting of the CI sound processor when patients filled out the questionnaires again and underwent the speech perception test in noise, again in the patient’s best-aided condition. Most patients used bilateral hearing aids prior to cochlear implantation; however, many patients stopped using their contralateral hearing aid after implantation.

### Participants

We recruited 134 adult patients referred for cochlear implantation at the Kuopio University Hospital from January 1, 2018 to December 31, 2020. We excluded patients referred for cochlear implantation because of single-sided deafness or referred for sequential bilateral implantation. Other exclusion criteria were a diagnosis of dementia or neurological or other health conditions that severely impair vision or mobility (as judged by the study physician). Patients who, despite their agreement to participate, did not respond to either the preoperative or to one of the postoperative questionnaires were also excluded. A total of 118 patients were included in the analyses. Patient demographics, preoperative pure-tone averages and surgical data are summarized in Table 1.

**TABLE 1:** Patient demographics and preoperative unaided pure-tone average (0.5–4 kHz).

	Mean	Median	Min	Max	SD
Age (years)	62.2	66.4	18	88	29.5

**Preoperative PTA_(0.5–4 kHz)_ (dB HL)**					
BEHL	80.5	81.9	33.8	110	18.0
WEHL	93.5	87.5	43.8	110	13.8

**Etiology of hearing loss**		**(*n*)**		**(*n*)**	

Unknown		66	**Sex**		
Meniere’s disease		20	Female	53	
Otosclerosis		6	Male	65	
Congenital SNHL		17			
NSSNHL		4	**Ear**		
SSNHL		2	Right	66	
Other		3	Left	52	

*BEHL, better ear hearing level; WEHL, worse ear hearing level; SNHL, sensorineural hearing loss; NSSNHL, non-syndromic sensorineural hearing loss; and SSNHL, syndromic sensorineural hearing loss.*

### Tests

#### The Finnish Nijmegen Cochlear Implant Questionnaire

The NCIQ comprises 60 questions divided into six subdomains of 10 questions each: basic sound perception (BSP), advanced sound perception (ASP), speech production (SPr), SE, activity (ACT), and SI. The answers to the questions were provided on a 5-point Likert scale (never, rarely, sometimes, often, and always) and scored with values of 0, 25, 50, 75, and 100. Participants also had the possibility to answer: “I don’t know” or “not applicable” to a question. These responses led to the exclusion of the question. The final score was the average of all responses and could range from 0 to 100. A higher score represented higher functioning. Separate scores were calculated for each NCIQ subdomain. Because the study was conducted with a Finnish population and an official Finnish translation of the NCIQ does not exist, the NCIQ was custom translated into Finnish.

#### The Finnish Speech, Spatial, and Qualities of Hearing Questionnaire

The SSQ comprises 49 questions divided into three subdomains: Speech Perception (SP), Spatial Hearing (SH) and other qualities of Hearing (SQ). The answers were provided on an 11-point Likert scale, ranging from 0 to 10. Answer scores were averaged for a final score and could range from 0 to 10. Separate scores were calculated for each subdomain and for an overall score. Because the study was conducted with a Finnish population and an official Finnish translation of the SSQ does not exist, we adapted the SSQ to Finnish culture and language.

#### The Finnish Matrix Sentence Test

The Finnish Matrix Sentence Test (FMST) was used as the SiN test. The FMST uses semantically unpredictable five-word sentences arranged in 20-item test lists (Dietz et al., 2014). The FMST has been validated in CI patients and has been found to be sensitive to subtle changes in hearing performance in normal-hearing participants as well as in CI recipients (Dietz et al., 2014, 2015). The clinical test protocol has been described in detail by Dietz et al. (2015). The background noise consisted of a stationary speech-shaped noise generated from the speech material and was presented at a fixed level of 65 dB SPL. The level of the speech signal changed adaptively to converge to each patient’s individual speech reception threshold in noise (SRTN), which is the signal-to-noise ratio (SNR) at which the patient recognizes 50% of the test items correctly. A total of three test lists were presented. The first list was always presented at a fixed SNR of +10 dB (i.e., signal 75 dB SPL, noise 65 dB SPL). The second and third test lists were administered with the adaptive measurement procedure. Only the third list was used as SRTN outcome measure. In patients with very poor hearing (defined here as those who scored <70% at +10 dB SNR), adaptive SRT measurements are not reliable; these patients thus did not undergo adaptive measurements (Dietz et al., 2015; Dingemanse and Goedegebure, 2019). In these cases, we defined a threshold of +5 dB SNR. This procedure resulted in two SiN measurements: (1) perceptual accuracy (in percent) at SNR +10 dB as measured by List 1 and (2) the SNR at 50% perceptual accuracy, i.e., the SRTN as measured by List 3.

#### Ethical Considerations

All patients were informed about the study aims and gave their written informed consent. The study complied with the Declaration of Helsinki on biomedical research involving human participants and received ethical approval from the Research Ethics Committee of the Northern Savo Hospital District (1327/2018).

#### Data Analysis and Statistics

Descriptive statistics are reported in the form of expected marginal means and 95% confidence intervals from univariate changes across the three time points (preoperative, postoperative 6 months, and postoperative 12 months) for all SiN results (+10 dB SNR and SRTN) and PROMs (NCIQ and SSQ). We also report *p*-values for the tests of differences between time points.

For adaptive SRT measurements in noise (SRTN) we used a Tobit model to account for censoring (Greene, 2003). Censoring refers to a situation in which we do not know the true value of a datapoint, or the observed value is too imprecise for values at or below a threshold and we only know that the true observation was lower than the threshold. Using this model was necessary because we used set values of +5 dB SNR for some of the participants, which then led to inaccuracies in statistical estimates. As a result, we adjusted the SRTN measurements as a left-censored normally distributed variable.

Longitudinal changes were assessed using a univariate and a bivariate latent change score model from the latent change score model framework (McArdle, 2009). In this model, latent variables represent individual changes occurring between time point pairs while adjusting for the baseline measurement. Supplementary Figures 1, 2 illustrate how these changes were modeled statistically. This model can be seen as an extension of the paired *t*-test over multiple time points (Coman et al., 2013) with the option to relax the assumptions of traditional models of change. We centered all time points on the baseline, i.e., preoperative mean, and thus, the unadjusted means of the change-variables correspond to baseline-adjusted paired *t*-tests for the changes. For univariate assessments we report baseline-adjusted standardized variances of change to enable assessment of change in variability over time. Standardized covariances between change variables are the residual correlations adjusted for baseline measurement. For bivariate assessment of change we report baseline-adjusted standardized covariances across SiN results and across the NCIQ BSP subdomain, NCIQ ASP subdomain, SSQ total, and SSQ subdomain scores. We also computed the unadjusted correlations of SSQ total and subdomain scores with the NCIQ subdomain scores. Using the R programming environment (R Core Team, 2020), mixed model parameters were estimated with package nlme (version 3.1-148) and marginal means and pairwise tests were computed with package emmeans (version 1.5.1, Pinheiro et al., 2020). Change score modeling was conducted in Mplus (version 7.4, Muthén and Muthén, (1998–2015)). Bivariate correlations were estimated and tested with the stats package in R (Length, 2020).

## Results

### Effect of Cochlear Implantation on Speech Perception in Noise and Patient-Reported Outcomes Measures

**FIGURE 1:**
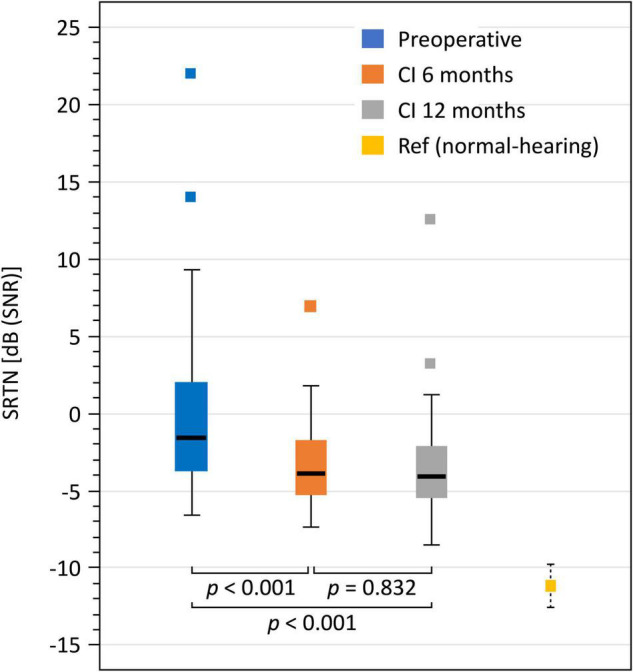
Boxplots for preoperative, 6 and 12 months SRTN including normal-hearing reference mean and 95% confidence interval.

The mean SiN scores at +10 dB SNR improved significantly from 76% preoperatively to 87 and 90% at 6 and 12 month follow-ups, respectively. Both improvements (pre- to 6 months postoperative and 6–12 months postoperative) were statistically significant (Table 2). The mean SRTNs improved significantly from −0.8 dB SNR preoperatively to −3.7 and −3.8 dB SNR at the 6 and 12 month follow-up, respectively, (Figure 1).

**TABLE 2:** Unstandardized means of change and regression coefficients for preoperative adjustment of change scores, and standardized covariance parameters in univariate latent change score models.

	Means						Regression coefficients					
	μ_△_PO−6m__			μ_△_6−12m__			Pre-op → Δ_PO–6 m_			Pre-op → Δ_6–12 m_		
Variable	Est	SE	*p*	Est	SE	*p*	Est	SE	*p*	Est	SE	*p*
SNR+10 dB	17.02	1.69	**<0.001**	2.95	1.40	**0.035**	–0.79	0.05	**<0.001**	–0.02	0.04	0.616
SRT	–2.61	0.24	**<0.001**	–0.42	0.19	**0.027**	–0.66	0.08	**<0.001**	0.11	0.07	0.097
NCIQ BSP	22.45	1.49	**<0.001**	0.15	1.44	0.918	–0.70	0.07	**<0.001**	0.04	0.07	0.552
NCIQ ASP	17.24	1.54	**<0.001**	–0.04	1.39	0.978	–0.80	0.08	**<0.001**	0.14	0.07	0.059
NCIQ SPr	7.86	1.49	**<0.001**	1.40	1.41	0.321	–0.53	0.08	**<0.001**	–0.03	0.08	0.658
NCIQ SE	13.76	1.66	**<0.001**	1.65	1.35	0.221	–0.61	0.10	**<0.001**	0.03	0.09	0.764
NCIQ ACT	18.24	2.10	**<0.001**	2.59	1.93	0.179	–0.69	0.10	**<0.001**	0.10	0.10	0.292
NCIQ SI	19.18	1.94	**<0.001**	–5.39	1.55	**0.001**	–0.56	0.09	**<0.001**	0.02	0.07	0.762
SSQ total	1.89	0.18	**<0.001**	0.00	0.17	1.000	–0.51	0.12	**<0.001**	0.07	0.11	0.541
SSQ SP	2.11	0.22	**<0.001**	–0.09	0.17	0.619	–0.60	0.13	**<0.001**	0.04	0.11	0.707
SSQ SH	1.84	0.21	**<0.001**	–0.26	0.21	0.220	–0.54	0.12	**<0.001**	0.24	0.12	0.055
SSQ SQ	1.77	0.19	**<0.001**	0.20	0.19	0.313	–0.56	0.11	**<0.001**	0.03	0.11	0.816

*SNR+10 dB, speech reception score; SRT, speech reception threshold; NCIQ, Nijmegen Cochlear Implant Questionnaire; SSQ, Speech, Spatial and Qualities of Hearing scale; NCIQ BSP, basic sound perception subdomain of NCIQ; NCIQ ASP, advanced sound perception subdomain of NCIQ; NCIQ SPr, speech production subdomain of NCIQ; NCIQ SE, self-esteem subdomain of NCIQ; NCIQ ACT, activity subdomain of NCIQ; NCIQ SI, social interactions subdomain of NCIQ; SSQ SP, speech perception subdomain of SSQ; SSQ SH, spatial hearing subdomain of SSQ; SSQ SQ, sound quality subdomain of SSQ; pre-op and PO, pre-operative; μ_i_, mean change in i; σ_x,y_, covariance between x and y; Δ_PO–6 m_, change score between pre-operative and 6 months values; Δ_6–12 m_, change score between 6 and 12 months values; Est, estimate; SE, standard error; p, p-value. Bold type face indicates p < 0.05; Standardization with respect to observed variables. Mean for pre-op measurement was centered to zero.*

**FIGURE 2:**
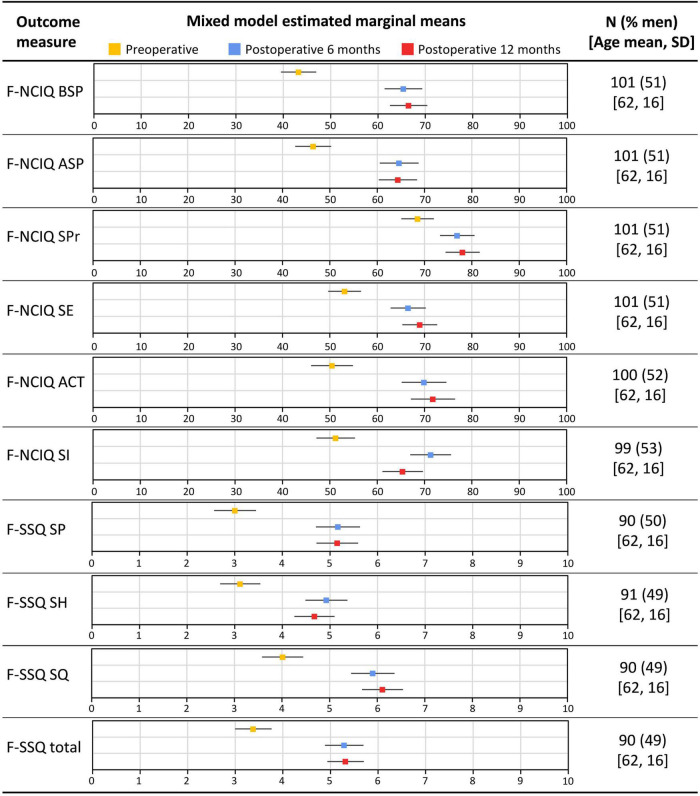
Estimated marginal means from unadjusted mixed models for the Nijmegen Cochlear Implant Questionnaire (NCIQ) subdomain scores and the Speech, Spatial and Qualities of Hearing scale (SSQ) subdomain and total score among CI recipients. BSP, basic sound perception; ASP, advanced sound perception; SPr, speech production; SE, self-esteem; ACT, activity; SI, social interactions; SP, speech perception; SH, spatial hearing; SQ, sound quality; and SD, standard deviation. Error bars indicate 95% confidence intervals.

The NCIQ and SSQ subdomain scores were analyzed using the univariate latent change score model. The results of estimated marginal means from unadjusted mixed models (rather than means of raw data) are shown in Figure 2. Table 2 shows the statistical results. For the NCIQ we observed significant improvements from preoperative testing to the 6 and 12 month follow-ups for all subdomains. We observed no additional significant improvements between the 6 and 12 month follow-ups. However, a slight but statistically significant decline (*p* = 0.001) was observed in the subdomain “social interaction.” For the SSQ scores we observed a statistically significant increase from preoperative testing to the 6 month follow-up for all scores. There was no further statistically significant improvement in any of the SSQ subdomains between 6 and 12 month follow-up points.

Table 2 also shows the regression coefficients for the preoperative adjustment of change scores. Specifically, this analysis shows that poorer preoperative SiN scores at +10 dB SNR and SRTN as well as PROM (NCIQ and SSQ) scores were significantly associated with larger improvements at 6 months follow-up. However, there was no association between the preoperative values and the change occurring between 6 and 12 months for any of the outcome measures.

### Covariance Analysis Between Speech Perception in Noise and Patient-Reported Outcomes Measures

The standardized covariance parameters between SiN scores at constant +10 dB SNR and SRTN as well as PROMs as calculated by the bivariate latent change score model are presented in Table 3. Significant positive covariances were found preoperatively between SiN perception scores at +10 dB SNR and the NCIQ BSP subdomain (*p* = 0.001) total SSQ and SSQ SP subdomain (both *p* ≤ 0.001). Significant negative covariances were found preoperatively between the SiN perception scores at +10 dB SNR and the NCIQ ASP and SSQ SH subdomains (both *p* < 0.001). In terms of change scores (preoperative to 6 months follow-up) significant positive covariances were found between the changes in SiN score at +10 dB SNR and the SSQ total (*p* = 0.012) and between SiN score and the SSQ SQ subdomain (*p* = 0.004). In addition, the analyses showed significant covariances between the change scores of SSQ SQ and SiN scores at constant +10 dB SNR 6 month postoperative in both directions of prediction.

**TABLE 3:** Standardized covariances between Speech-in-noise (SiN) scores and patient reported outcomes (PROMs) (bivariate latent change score models).

Variables		σ_Y_pre−op_,X_pre−op__			σ_△Y_PO−6m_,△X_PO−6m__			σ_△X_PO−6m_,△Y_6−12m__			σ_△Y_PO−6m_,△X_6−12m__			σ_△Y_6−12m_,△X_6−12m__		
*X*	*Y*	Est	SE	*p*	Est	SE	*p*	Est	SE	*p*	Est	SE	*p*	Est	SE	*p*
SNR+10 dB	NCIQ BSP	0.36	0.09	**0.001**	0.20	0.13	0.134	0.01	0.13	0.911	0.04	0.15	0.788	–0.13	0.14	0.376
	NCIQ ASP	–0.53	0.08	**<0.001**	–0.04	0.16	0.788	–0.09	0.22	0.670	0.18	0.19	0.348	–0.16	0.30	0.592
	SSQ total	0.49	0.08	**<0.001**	0.34	0.12	**0.012**	–0.08	0.12	0.511	0.18	0.15	0.249	–0.01	0.14	0.923
	SSQ SP	0.36	0.10	**0.001**	0.26	0.14	0.077	–0.20	0.13	0.144	–0.01	0.17	0.935	0.19	0.15	0.225
	SSQ SH	–0.52	0.08	**<0.001**	–0.02	0.15	0.904	–0.19	0.14	0.173	–0.03	0.16	0.868	0.27	0.14	0.066
	SSQ SQ	0.14	0.10	0.189	0.43	0.13	**0.004**	–0.35	0.13	**0.017**	–0.17	0.17	0.305	0.31	0.14	**0.039**
SRT	NCIQ BSP	–0.50	0.08	**<0.001**	–0.03	0.15	0.853	–0.13	0.18	0.469	0.04	0.19	0.813	–0.06	0.23	0.800
	NCIQ ASP	0.35	0.10	**0.002**	0.22	0.14	0.139	–0.25	0.13	0.068	–0.07	0.16	0.665	0.26	0.14	0.072
	SSQ total	–0.49	0.08	**<0.001**	–0.04	0.16	0.824	0.11	0.21	0.601	0.01	0.18	0.975	–0.29	0.21	0.192
	SSQ SP	–0.21	0.10	**0.049**	–0.17	0.16	0.304	0.08	0.22	0.714	–0.04	0.19	0.839	–0.23	0.23	0.335
	SSQ SH	0.39	0.10	**<0.001**	0.07	0.15	0.645	0.07	0.21	0.747	–0.06	0.17	0.735	–0.27	0.24	0.264
	SSQ SQ	–0.60	0.07	**<0.001**	–0.10	0.17	0.571	0.26	0.24	0.281	0.09	0.18	0.625	–0.36	0.21	0.111

*SNR+10 dB, speech reception score; SRT, speech reception threshold; NCIQ, Nijmegen Cochlear Implant Questionnaire; SSQ, Speech, Spatial and Qualities of Hearing scale; NCIQ BSP, basic sound perception subdomain of NCIQ; NCIQ ASP, advanced sound perception subdomain of NCIQ; SSQ SP, speech perception subdomain of SSQ; SSQ SH, spatial hearing subdomain of SSQ; SSQ SQ, sound quality subdomain of SSQ; pre-op and PO, pre-operative; ΔY_PO–6 m_, change score between pre-operative and 6 months value for variable Y; ΔY_6–12 m_, change score between 6 and 12 months value for variable Y, σ_x,y_, covariance between x and y. Bold type face indicates p < 0.05.*

The standardized covariance parameters between SRTN results and PROMs presented a very similar picture. There were statistically significant negative covariances preoperatively between SRTN and the NCIQ BSP subdomain (*p* < 0.001), SSQ total score (*p* < 0.001), SSQ SP subdomain (*p* = 0.049), and SSQ SQ subdomain (*p* < 0.001). There were statistically significant positive covariances preoperatively between SRTN and the NCIQ ASP (*p* = 0.002) and SSQ SH subdomains (*p* < 0.001; N.B. a negative SRTN indicates better performance). There were no statistically significant covariances between the SRTN and PROM changes from preoperative scores to 6 month postoperative scores.

### Correlations Between Nijmegen Cochlear Implant Questionnaire Subdomains With Speech, Spatial, Qualities of Hearing Questionnaire

Between the SSQ total score and each of the NCIQ subdomains there were statistically significant, moderate-to-strong correlations within all the time points (*r* = 0.42–0.69, *p* < 0.001) as shown in Table 4. The correlations were strongest between SSQ total and the NCIQ ASP subdomain and weakest between SSQ total and the NCIQ SE subdomain.

**TABLE 4:** Pearson correlation for associations between the SSQ total score and component tests of the NCIQ among all participants.

	Unadjusted model			
			95% CI	
	*n*	*r*	Lower	Upper
**Pre-operative**				
SSQ total vs. NCIQ BSP	100	0.65	0.52	0.75
SSQ total vs. NCIQ ASP	101	0.68	0.56	0.77
SSQ total vs. NCIQ SPr	101	0.48	0.32	0.62
SSQ total vs. NCIQ SE	101	0.47	0.31	0.61
SSQ total vs. NCIQ ACT	98	0.53	0.37	0.66
SSQ total vs. NCIQ SI	100	0.55	0.40	0.68
**6 months**				
SSQ total vs. NCIQ BSP	78	0.53	0.35	0.67
SSQ total vs. NCIQ ASP	78	0.63	0.47	0.75
SSQ total vs. NCIQ SPr	78	0.45	0.25	0.61
SSQ total vs. NCIQ SE	78	0.42	0.22	0.59
SSQ total vs. NCIQ ACT	78	0.55	0.37	0.69
SSQ total vs. NCIQ SI	78	0.54	0.36	0.68
**12 months**				
SSQ total vs. NCIQ BSP	83	0.62	0.46	0.73
SSQ total vs. NCIQ ASP	83	0.69	0.56	0.79
SSQ total vs. NCIQ SPr	83	0.57	0.41	0.70
SSQ total vs. NCIQ SE	83	0.55	0.38	0.68
SSQ total vs. NCIQ ACT	83	0.66	0.52	0.77
SSQ total vs. NCIQ SI	83	0.63	0.48	0.74

*SSQ total, Speech, Spatial and Qualities of Hearing scale; NCIQ, Nijmegen Cochlear Implant Questionnaire; NCIQ BSP, basic sound perception subdomain of NCIQ; NCIQ ASP, advanced sound perception subdomain of NCIQ; NCIQ SPr, speech production subdomain of NCIQ; NCIQ SE, self-esteem subdomain of NCIQ; NCIQ ACT, activity subdomain of NCIQ; and NCIQ SI, social interactions subdomain of NCIQ; n = sample size available for analysis, r = Pearson correlation, and CI = confidence interval. For all correlations p < 0.001.*

### Correlation Between Speech Perception in Noise and Patient-Reported Outcomes Measures

Statistically significant correlations between SiN measures (+10 dB SNR and SRTN) and PROMs (NCIQ and SSQ) scores within a time point of measurement were most evident preoperatively (Table 5). At the 6 month follow-up, the only statistically significant correlation was between the SiN scores at +10 dB SNR and the NCIQ ASP subdomain. At the 12 month follow-up, the only statistically significant correlations were between SRTN and SSQ total score, and between SiN at +10 dB SNR and SSQ total.

**TABLE 5:** Unadjusted Pearson correlation for associations between SiN perception (+10 dB SNR and SRTN) results and the NCIQ and SSQ among all participants.

	Unadjusted				
			95% CI		
	*n*	*r*	Lower	Upper	*p*-value
**Pre-operative**					
SNR+10 dB vs. NCIQ BSP	103	0.35	0.17	0.51	**<0.001**
SNR+10 dB vs. NCIQ ASP	104	0.46	0.29	0.60	**<0.001**
SNR+10 dB vs. SSQ total	78	0.41	0.21	0.58	**<0.001**
SNR+10 dB vs. SSQ SP	59	0.08	–0.18	0.33	0.561
SNR+10 dB vs. SSQ SH	58	–0.01	–0.26	0.25	0.959
SNR+10 dB vs. SSQ SQ	58	–0.04	–0.29	0.23	0.794
SRT vs. NCIQ BSP	85	–0.52	–0.66	–0.34	**<0.001**
SRT vs. NCIQ ASP	85	–0.46	–0.61	–0.28	**<0.001**
SRT vs. SSQ total	65	–0.46	–0.63	–0.24	**<0.001**
SRT vs. SSQ SP	59	–0.03	–0.29	0.22	0.798
SRT vs. SSQ SH	58	–0.12	–0.36	0.15	0.385
SRT vs. SSQ SQ	58	–0.16	–0.40	0.10	0.223
**6 months**					
SNR+10 dB vs. NCIQ BSP	53	0.17	–0.10	0.42	0.216
SNR+10 dB vs. NCIQ ASP	53	0.28	0.01	0.51	**0.040**
SNR+10 dB vs. SSQ total	50	0.11	–0.17	0.38	0.439
SNR+10 dB vs. SSQ SP	33	–0.07	–0.40	0.28	0.705
SNR+10 dB vs. SSQ SH	32	–0.05	–0.39	0.30	0.782
SNR+10 dB vs. SSQ SQ	33	–0.06	–0.40	0.29	0.739
SRT vs. NCIQ BSP	48	–0.05	–0.33	0.24	0.731
SRT vs. NCIQ ASP	48	–0.11	–0.39	0.18	0.440
SRT vs. SSQ total	46	–0.09	–0.37	0.21	0.555
SRT vs. SSQ SP	33	–0.07	–0.40	0.28	0.705
SRT vs. SSQ SH	32	–0.05	–0.39	0.30	0.782
SRT vs. SSQ SQ	33	–0.06	–0.40	0.29	0.739
**12 months**					
SNR+10 dB vs. NCIQ BSP	62	0.08	–0.17	0.32	0.531
SNR+10 dB vs. NCIQ ASP	62	0.30	0.06	0.51	**0.017**
SNR+10 dB vs. SSQ total	61	–0.01	–0.26	0.24	0.943
SNR+10 dB vs. SSQ SP	32	0.14	–0.22	0.46	0.456
SNR+10 dB vs. SSQ SH	32	–0.24	–0.54	0.12	0.190
SNR+10 dB vs. SSQ SQ	32	0.01	–0.34	0.36	0.963
SRT vs. NCIQ BSP	53	–0.10	–0.36	0.17	0.473
SRT vs. NCIQ ASP	53	–0.18	–0.43	0.10	0.206
SRT vs. SSQ total	52	–0.31	–0.53	–0.04	**0.028**
SRT vs. SSQ SP	32	0.14	–0.22	0.46	0.456
SRT vs. SSQ SH	32	–0.24	–0.54	0.12	0.190
SRT vs. SSQ SQ	32	0.01	–0.34	0.36	0.963

*SRT, speech reception threshold; SNR+10 dB, speech reception score; NCIQ, Nijmegen Cochlear Implant Questionnaire; NCIQ BSP, basic sound perception subdomain of NCIQ; NCIQ ASP, advanced sound perception subdomain of NCIQ; SSQ total, Speech Spatial and Qualities of Hearing scale total score; SSQ SP, speech perception subdomain of SSQ; SSQ SH, spatial hearing subdomain of SSQ; SSQ SQ, sound quality subdomain of SSQ; n = sample size available for analysis; r = Pearson correlation; and CI = confidence interval. Bold type face indicates p < 0.05.*

## Discussion

When assessing the clinical outcomes of cochlear implantation, the focus normally lies almost exclusively on performance-based behavioral outcome measures such as speech perception. However, these measures often correlate poorly with perceived benefits (Capretta and Moberly, 2016; McRackan et al., 2019; Vasil et al., 2020). As such, they do not fully address the ultimate goal of cochlear implantation, which is to improve the patients’ QoL by restoring their speech perception and providing them with adequate communication skills to fully resume spcial interaction and participation. Using additional PROMs to assess QoL and other relevant subdomains of functioning would help to rectify this; however, PROMs are rarely included in the clinical routine to assess the outcomes of cochlear implantation. One reason for the omission of PROMs in the assessment is the lack of adequate instruments that fulfill modern standards, i.e., are fully psychometrically validated for the patient group. Currently, most PROMs used for CI patients were originally developed for hearing aid users, who have a very different HL profile (mild to moderate HL) and a very different rehabilitation strategy (hearing aids). Instruments developed for a different population and a different rehabilitation strategy are unlikely to adequately assess the experiences of CI users. However, one PROM that was specifically developed for CI users is the NCIQ (Hinderrick et al., 2000).

In this study, we aimed to assess the patient-reported benefits of unilateral cochlear implantation in an unselected Finnish patient cohort of patients with bilateral HL, and compare it to behaviorally assessed speech perception scores. To assess patient-reported benefit we used the NCIQ, a PROM specifically developed for CI-users, and the SSQ. An additional challenge for our study was the lack of PROMs for the use with Finnish patients. Therefore, we adapted the NCIQ and the SSQ to Finnish culture and language.

We found significant improvements in SiN measures (both at a constant SNR of +10 dB and at SRTN), perceived activity limitations and QoL after cochlear implantation. When SiN is measured with a presentation level of +10 dB SNR, the level of the speech signal is so much higher than the noise level that this test condition can be likened to speech perception in quiet. With this in mind, the improvement in SiN scores measured at a constant SNR (+10 dB SNR) fits well with previous results that have shown robust improvements in speech perception in quiet after implantation (Mudery et al., 2017; Zwolan et al., 2020). The improvement we found for SRTN demonstrates that cochlear implantation is also effective in improving patients’ speech perception in complex listening environments. The SiN perception results in the present study are comparable to those reported in a previous study in a different cohort of patients at our institution (Dietz et al., 2015). The characteristics and reference values of speech audiometry differ across languages, which makes a direct comparison of the postoperative results in the international context difficult.

The patient-reported improvements show that implantation is not only effective in improving speech perception but also in alleviating activity limitations and improving QoL across a wider range of listening situations. Notably, the benefits measured with the NCIQ and SSQ in this study for a Finnish CI population are similar in magnitude to those recently reported in a systematic review by Andries et al. (2021). This similarity emerged despite possible variations across countries and languages: for example, differences in the indications for cochlear implantation between different countries and healthcare systems, or differences in perceived benefits across different languages. For tonal languages, for example, the impact of cochlear implantation may be more limited, as they rely significantly on the adequate reproduction of spectral and temporal cues, which current CI technology is unable to provide (Wei et al., 2004).

The fact that correlations between SiN results and PROMs decrease for postoperative measurements suggests that the two types of measures assess different aspects of functioning. It is possible that even though SiN tests (such as the FMST) simulate everyday listening situations more accurately than tests performed in quiet, they still fail to capture the manifold hearing environments of everyday life. Future studies will need to examine to what extent speech perception tests in simulated realistic acoustic environments are better able to capture everyday listening and whether this leads to higher correlations with PROMs postoperatively.

The present study demonstrates that the main improvements in speech perception and PROMs took place within the first 6 months postoperatively. This is in line with previous data, which have shown that the main improvements in outcome measures can be seen within the first 6 months after fitting of the sound processor (Lenarz et al., 2012; Zhang et al., 2015; Häußler et al., 2019). Although we observed an additional, statistically significant, improvement in the speech perception tests between 6 and 12 months, the magnitude of improvement was small and within the limits of the test and statistical sensitivity. These results highlight the importance of adequate care and sound processor fitting during the early months of rehabilitation.

There was a slight, but statistically significant, decline of scores in the SI subdomain of the NCIQ between 6 and 12 months postoperative measurements. Based on our clinical experience, we speculate that the decline in the SI subdomain between 6 and 12 months is due to the fact that increased SI after cochlear implantation (observed after 6 months of use) expose patients more often to complex listening situation, which then discloses the limitations of hearing with the CI resulting in a decrease of SI. In addition, patients often stop using their contralateral hearing aids after adaptation to the CI and also often inquire about the possibility of getting a second CI in their contralateral ear.

The regression coefficients showed that lower preoperative PROMs and speech perception results indicated greater changes 6 month postoperatively. This is expected, as CIs can reliably restore the patient’s functional hearing to an adequate performance level so that they can have a relaxed conversation in quiet surroundings. Therefore, patients with the most profound HL [i.e., patients who are not able to have any (relaxed) conversation] are more likely to perceive improvements in their hearing as more significant than patients with less severe loss. Patients with less severe HL usually experience problems in complex sound environments, in which the benefits of CIs can be more variable.

Looking at the correlation measures, we found a strong correlation between the NCIQ subdomains of BSP and ASP and the SSQ total score, and a moderate correlation between the remaining subdomains of the NCIQ and the SSQ total score. The SSQ assesses mainly activity limitations associated with hearing whereas the NCIQ is thought to focus more on participation restrictions associated with socioemotional factors. This suggests an existing interconnection between HL and socioemotional issues.

Only a few studies have investigated the relationships between PROMs and SiN measures (fixed SRN and SRTN), and found mainly weak correlations (Hirschfelder et al., 2008; Vasil et al., 2020; West et al., 2020). In the present study, we found statistically significant correlations for some subdomains exclusively at the preoperative assessment (see Table 5). Importantly, we found no clinically significant associations or correlations between SiN measures and PROMs for any of the follow-up evaluations, indicating that the cochlear implantation benefits were not fully captured by the SiN test. The baseline-adjusted covariances support this, with performance-based measures showing an association with the SSQ (and its subdomains) and NCIQ (BSP, ASP) only before the intervention. Although, we also found statistically significant covariances between SiN scores (at +10 dB SNR) and some PROMs subdomains postoperatively, these have to be interpreted with caution, as no corresponding significant covariances were observed between these subdomains and the SRTN, which is the more precise and more reliable measure of performance. Taken together, these results confirm that there are other hearing-related CI benefits which are not captured by auditory performance measured with current SiN tests.

## Limitations and Strengths of the Study

Several limitations associated with this study need attention. The original questions of the NCIQ and the SSQ were created by expert opinion, and the psychometric qualities of each question as well as of the questionnaires as a whole are still not fully understood. Further studies are required to fully evaluate these psychometric properties, both in terms of classical test theory (e.g., test-retest reliability, minimal relevant change) and also wider assessment along the COSMIN guidelines (Mokkink et al., 2010), and content and construct validity. Therefore, caution should be applied when interpreting these PROMs. However, as the magnitude of improvement after cochlear implantation measured in this study was substantial, it is beyond any reasonable doubt that these benefits exist.

The strengths of this study are the relatively large cohort of patients and its prospective, longitudinal design, which gives a less biased estimate of population measures than the more commonly used retrospective and cross-sectional designs. In addition, we not only report the change scores in patient-reported and behavioral outcome measures but have also investigated their association, as well as the relationship between baseline-adjusted changes at various time points.

## Conclusion

Cochlear implantation significantly improves speech perception, QoL (NCIQ), and self-assessed hearing capabilities (SSQ) in a cohort of unselected Finnish CI recipients. The main improvements were observed within the first 6 months after sound processor activation, indicating the importance of adequate early sound processor fitting. The results highlight the fact that cochlear implantation conveys benefits which go beyond those captured by performance-based outcome measures.

## Data Availability Statement

The raw data supporting the conclusions of this article will be made available by the authors, without undue reservation.

## Ethics Statement

The studies involving human participants were reviewed and approved by Research Ethics Committee of the Northern Savo Hospital District (1327/2018). The patients/participants provided their written informed consent to participate in this study.

## Author Contributions

AD, TW, MI-M, and PL contributed to conception and design of the study. PL and MI-M organized the database. TT performed the statistical analysis and designed the figures and tables. AD and AH wrote the first draft of the manuscript. PL, TW, and PM wrote sections of the manuscript. All authors contributed to manuscript revision, read, and approved the submitted version.

## Conflict of Interest

The authors declare that the research was conducted in the absence of any commercial or financial relationships that could be construed as a potential conflict of interest.

## Publisher’s Note

All claims expressed in this article are solely those of the authors and do not necessarily represent those of their affiliated organizations, or those of the publisher, the editors and the reviewers. Any product that may be evaluated in this article, or claim that may be made by its manufacturer, is not guaranteed or endorsed by the publisher.

## References

[B1] Andries E. Gilles A. Topsakal V. Vanderveken O. M. Van de Heyning P. Van Rompaey V. (2021). Review of quality of life assessments after cochlear implantation in older adults. *Audiol. Neurotol.* 26 61–75. 10.1159/000508433 32653882

[B2] Boisvert I. Reis M. Au A. Cowan R. Dowell R. C. (2020). Cochlear implantation outcomes in adults: a scoping review. *PLoS One* 15:e0232421. 10.1371/journal.pone.0232421 32369519 PMC7199932

[B3] Capretta N. R. Moberly A. C. (2016). Does quality of life depend on speech recognition performance for adult cochlear implant users? *Laryngoscope* 126 699–706. 10.1002/lary.25525 26256441

[B4] Coman E. N. Picho K. McArdle J. J. Villagra V. Dierker L. Iordache E. (2013). The paired t-test as a simple latent change score model. *Front. Psychol.* 4:738. 10.3389/fpsyg.2013.00738 24124419 PMC3794455

[B5] Dietz A. Buschermöhle M. Aarnisalo A. A. Vanhanen A. Hyyrynen T. Aaltonen O. (2014). The development and evaluation of the finnish matrix sentence test for speech intelligibility assessment. *Acta Otolaryngol.* 134 728–737. 10.3109/00016489.2014.898185 24807850

[B6] Dietz A. Buschermöhle M. Sivonen V. Willberg T. Aarnisalo A. A. Lenarz T. (2015). Characteristics and international comparability of the Finnish matrix sentence test in cochlear implant recipients. *Int. J. Audiol.* 54(Suppl. 2) 80–87. 10.3109/14992027.2015.1070309 26364512

[B7] Dingemanse G. Goedegebure A. (2019). Efficient adaptive speech reception threshold measurements using stochastic approximation algorithms. *Trends Hear.* 23:2331216520919199. 10.1177/2331216520919199 32425135 PMC7238302

[B8] Gatehouse S. Noble W. (2004). The speech, spatial and qualities of hearing scale (SSQ). *Int. J. Audiol.* 43 85–99. 10.1080/14992020400050014 15035561 PMC5593096

[B9] Gifford R. H. Shallop J. K. Peterson A. M. (2008). Speech recognition materials and ceiling effects: considerations for cochlear implant programs. *Audiol. Neurootol.* 13 193–205. 10.1159/000113510 18212519

[B10] Greene W. H. (2003). *Econometric Analysis*, 5th Edn. Upper Saddle River, NJ: Prentice Hall.

[B11] Häußler S. M. Knopke S. Wiltner P. Ketterer M. Gräbel S. Olze H. (2019). Long-term benefit of Unilateral cochlear implantation on quality of life and speech perception in bilaterally deafened patients. *Otol. Neurotol.* 40 e430–e440. 10.1097/MAO.0000000000002008 30870378

[B12] Hinderink J. B. Krabbe P. F. Van Den Broek P. (2000). Development and application of a health-related quality-of-life instrument for adults with cochlear implants: the Nijmegen cochlear implant questionnaire. *Otolaryngol Head Neck Surg* 123 756–765. 11112975 10.1067/mhn.2000.108203

[B13] Hirschfelder A. Gräbel S. Olze H. (2008). The impact of cochlear implantation on quality of life: the role of audiologic performance and variables. *Otolaryngol. Head Neck Surg.* 138 357–362. 10.1016/j.otohns.2007.10.019 18312885

[B14] Holden L. K. Finley C. C. Firszt J. B. Holden T. A. Brenner C. Potts L. G. (2013). Factors affecting open-set word recognition in adults with cochlear implants. *Ear Hear.* 34 342–360. 10.1097/AUD.0b013e3182741aa7 23348845 PMC3636188

[B15] Kramer S. E. Kapteyn T. S. Festen J. M. Kramer S. E. (1998). The self-reported handicapping effect of hearing disabilities. *Audiology* 37 302–312. 10.3109/00206099809072984 9776207

[B16] Lenarz M. Sönmez H. Joseph G. Büchner A. Lenarz T. (2012). Long-term performance of cochlear implants in postlingually deafened adults. *Otolaryngol. Head Neck Surg.* 147 112–118. 10.1177/0194599812438041 22344289

[B17] Length R. (2020). *emmeans: Estimated Marginal Means, Aka Least-Squares Means. R Package Version 1.5.1.* Available online at: https://CRAN.R-project.org/package=emmeans (accessed June 23, 2021).

[B18] Lin F. R. Metter E. J. O’Brien R. J. Resnick S. M. Zonderman A. B. Ferrucci L. (2011). Hearing loss and incident dementia. *Arch. Neurol.* 68 214–220. 10.1001/archneurol.2010.362 21320988 PMC3277836

[B19] Loughrey D. G. Kelly M. E. Kelley G. A. Brennan S. Lawlor B. A. (2018). Association of age-related hearing loss with cognitive function, cognitive impairment, and dementia: a systematic review and meta-analysis. *JAMA Otolaryngol. Head Neck Surg.* 144 115–126. 10.1001/jamaoto.2017.2513 29222544 PMC5824986

[B20] McArdle J. J. (2009). Latent variable modeling of differences and changes with longitudinal data. *Annu. Rev. Psychol.* 60 577–605. 10.1146/annurev.psych.60.110707.163612 18817479

[B21] McRackan T. R. Hand B. N. Velozo C. A. Dubno J. R. (2019). Association of demographic and hearing-related factors with cochlear implant-related quality of life. *JAMA Otolaryngol. Head Neck Surg.* 145 422–430. 10.1001/jamaoto.2019.0055 30896742 PMC6537791

[B22] Mertens G. Andries E. Claes A. J. Topsakal V. Van de Heyning P. Van Rompaey V. (2020). Cognitive improvement after cochlear implantation in older adults with severe or profound hearing impairment: a prospective, longitudinal, controlled, multicenter Study. *Ear Hear.* 42 606–614. 10.1097/AUD.0000000000000962 33055579 PMC8088820

[B23] Mokkink L. B. Terwee C. B. Patrick D. L. Alonso J. Stratford P. W. Knol D. L. (2010). The COSMIN study reached international consensus on taxonomy, terminology, and definitions of measurement properties for health-related patient-reported outcomes. *J. Clin. Epidemiol.* 63 737–745. 10.1016/j.jclinepi.2010.02.006 20494804

[B24] Mudery J. Francis R. McCary H. Jacob A. (2017). Older individuals meeting medicare cochlear iplant candidacy criteria in noise but not in quiet: are these patients improved by surgery? *Otol. Neurotol.* 38 187–191. 27832005 10.1097/MAO.0000000000001271

[B25] Muthén L. K. Muthén B. O. (1998–2015). *Mplus: Version 7.4.* Los Angeles, CA: Muthén & Muthén.

[B26] Ottaviani F. Iacona E. Sykopetrites V. Schindler A. Mozzanica F. (2016). Cross-cultural adaptation and validation of the Nijmegen cochlear implant questionnaire into Italian. *Eur. Arch. Otorhinolaryngol.* 273 2001–2007. 10.1007/s00405-015-3765-8 26324881

[B27] Pinheiro J. Bates D. DebRoy S. Deepayan S. (2020). *nlme: Linear and Nonlinear Mixed Effects Models. R Package Version 3.1-148.* Available online at: https://CRAN.R-project.org/package=nlme (accessed June 23, 2021).

[B28] R Core Team (2020). *R: A Language and Environment for Statistical Computing.* Vienna: R Foundation for Statistical Computing.

[B29] Sanchez-Cuadrado I. Gavilan J. Perez-Mora R. Muñoz E. Lassaletta L. (2015). Reliability and validity of the Nijmegen cochlear implant questionnaire in Spanish. *Eur. Arch. Otorhinolaryngol.* 272 1621–1625. 10.1007/s00405-014-2983-9 24609736

[B30] Santos N. P. D. Couto M. I. V. Martinho-Carvalho A. C. (2017). Nijmegen Cochlear Implant Questionnaire (NCIQ): translation, cultural adaptation, and application in adults with cochlear implants. *Codas* 29:e20170007. 10.1590/2317-1782/20172017007 29236905

[B31] Vasil K. Lewis J. Tamati T. Ray C. Moberly A. (2020). How does quality of life relate to auditory abilities? A subitem analysis of the Nijmegen cochlear implant questionnaire. *J. Am. Acad. Audiol.* 31 292–301. 31580803 10.3766/jaaa.19047 PMC7103517

[B32] Vermiglio A. J. Soli S. D. Freed D. J. Fisher L. M. (2012). The relationship between high-frequency pure-tone hearing loss, hearing in noise test (HINT) thresholds, and the articulation index. *J. Am. Acad. Audiol.* 23 779–788. 10.3766/jaaa.23.10.4 23169195

[B33] Wei C. Cao K. Zeng F. (2004). Mandarin tone recognition in cochlear-implant subjects. *Hear. Res.* 197 87–95. 10.1016/j.heares.2004.06.002 15504607

[B34] West N. C. Kressner A. A. Baungaard L. H. Sandvej M. G. Bille M. Cayé-Thomasen P. (2020). Nordic results of cochlear implantation in adults: speech perception and patient reported outcomes. *Acta Otolaryngol.* 140 939–947. 10.1080/00016489.2020.1816656 32957807

[B35] World Health Organization [WHO] (2021). *World Report on Hearing.* Geneva: World Health Organization. Licence: CC BY-NC-SA 3.0 IGO.

[B36] World Health Organsation [WHO] (2001). *International Classification of Functioning, Disability and Health: ICF.* Geneva: World Health Organization.

[B37] Zhang J. Tyler R. Ji H. Dunn C. Wang N. Hansen M. (2015). Speech, spatial and qualities of hearing scale (SSQ) and spatial hearing questionnaire (SHQ) changes over time in adults with simultaneous cochlear implants. *Am. J. Audiol.* 24 384–397. 10.1044/2015_AJA-14-0074 25934950 PMC4659416

[B38] Zwolan T. Kallogjeri D. Firszt J. Buchman C. (2020). Assessment of cochlear implants for adult medicare beneficiaries aged 65 years or older who meet expanded indications of open-set sentence Recognition: a multicenter nonrandomized clinical trial. *JAMA Otoloaryngol. Head Neck Surg.* 146 933–941. 10.1001/jamaoto.2020.2286 PMC7453340 32857106

