# Supplementary figures and images for: The Effectiveness of Unilateral Cochlear Implantation on Performance-Based and Patient-Reported Outcome Measures in Finnish Recipients
Source: Front Neurosci. 2022 Jun 6;16:786939. doi: 10.3389/fnins.2022.786939 (PMC9207276; doi:10.3389/fnins.2022.786939)

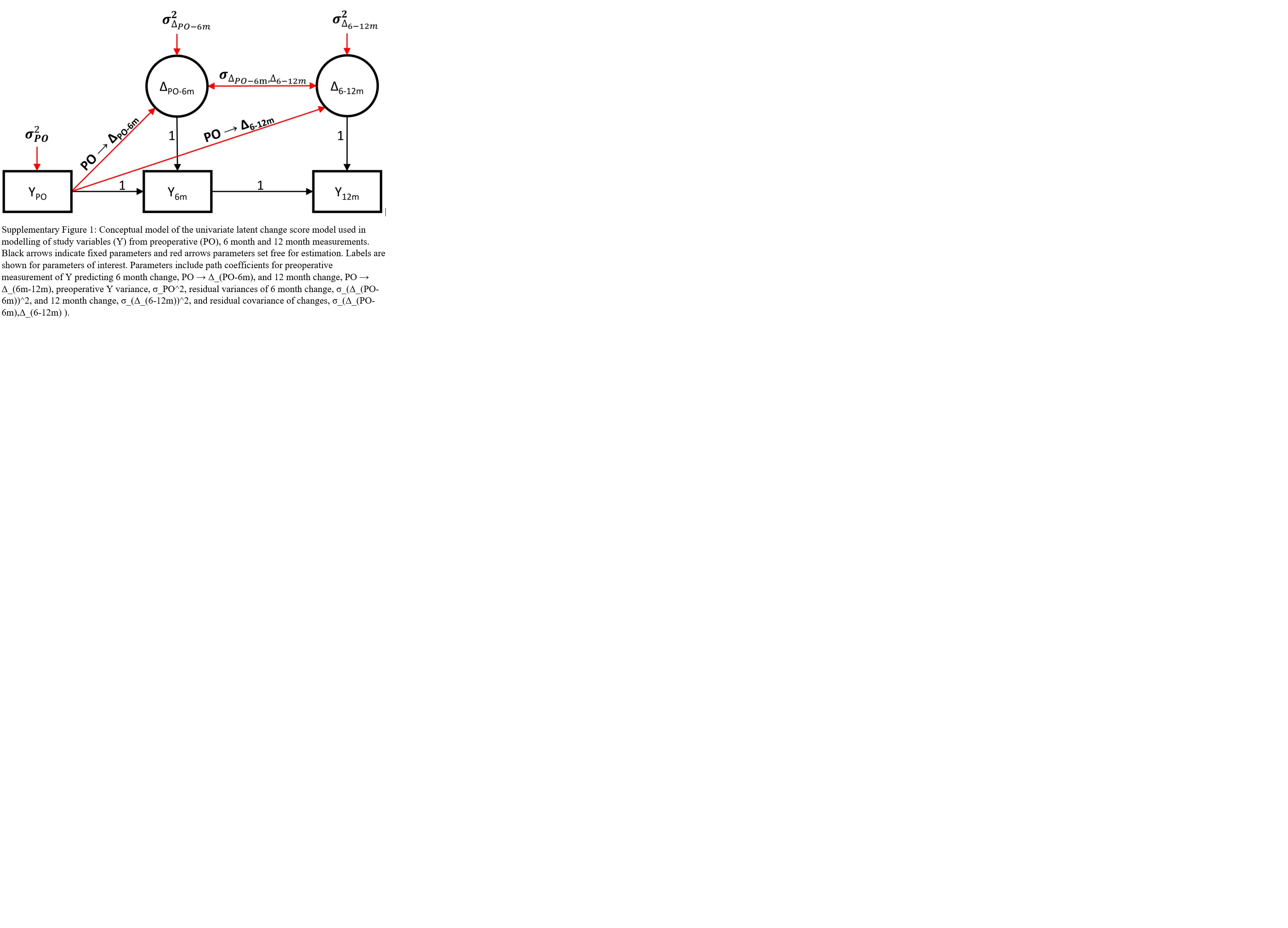

Supplement: Supplementary file 1 [file Image_1.JPEG]

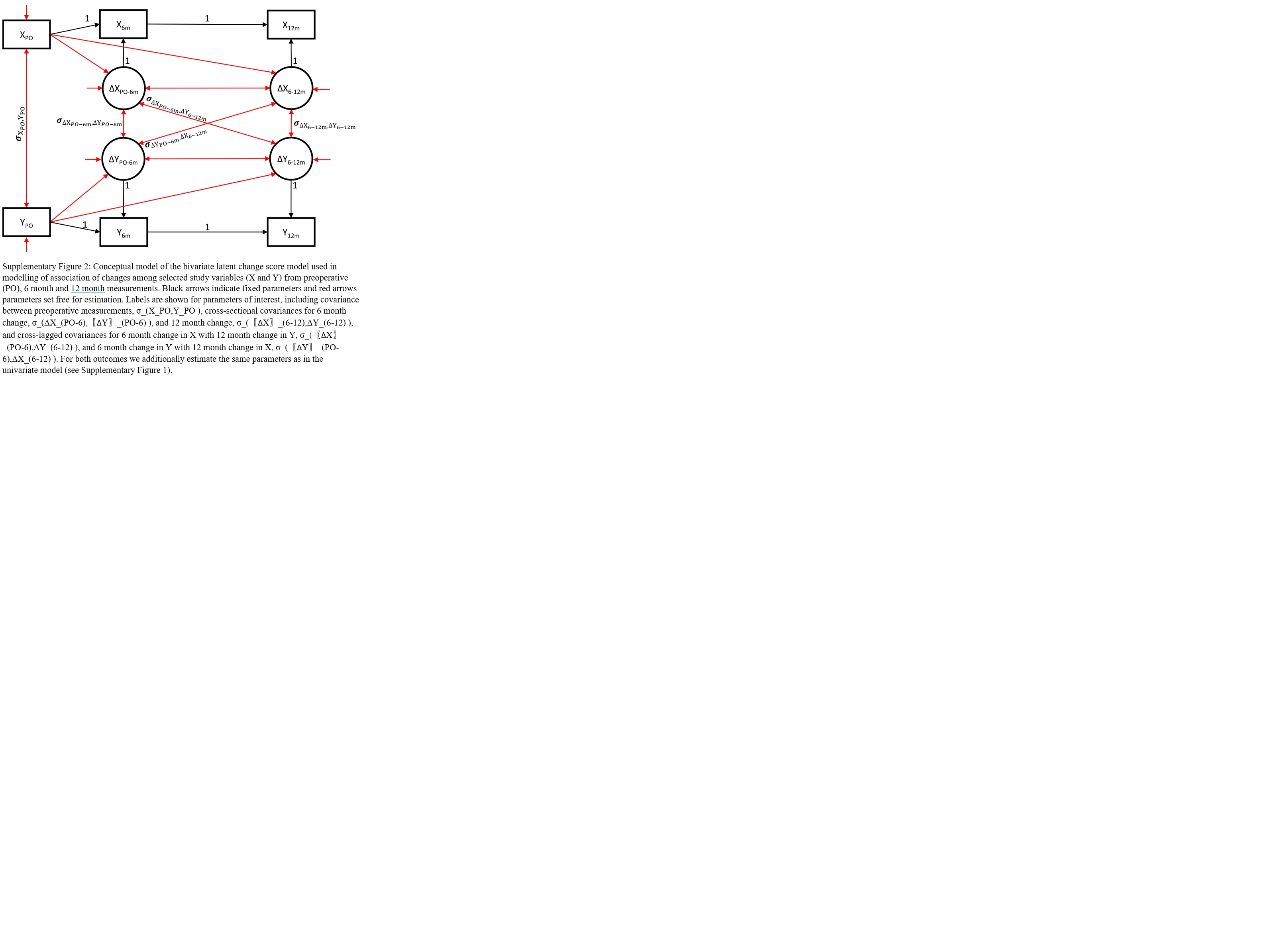

Supplement: Supplementary file 2 [file Image_2.JPEG]
